# Mining influential genes based on deep learning

**DOI:** 10.1186/s12859-021-03972-5

**Published:** 2021-01-22

**Authors:** Lingpeng Kong, Yuanyuan Chen, Fengjiao Xu, Mingmin Xu, Zutan Li, Jingya Fang, Liangyun Zhang, Cong Pian

**Affiliations:** 1 College of Agriculture, Nanjing Agricultural University, Jiangsu, 210095 Nanjing China; 2 Department of Mathematics, College of Science, Nanjing Agricultural University, Nanjing, 210095 China

**Keywords:** Landmark genes, Deep learning, AutoEncoder, DeepLIFT

## Abstract

**Background:**

Currently, large-scale gene expression profiling has been successfully applied to the discovery of functional connections among diseases, genetic perturbation, and drug action. To address the cost of an ever-expanding gene expression profile, a new, low-cost, high-throughput reduced representation expression profiling method called L1000 was proposed, with which one million profiles were produced. Although a set of ~ 1000 carefully chosen landmark genes that can capture ~ 80% of information from the whole genome has been identified for use in L1000, the robustness of using these landmark genes to infer target genes is not satisfactory. Therefore, more efficient computational methods are still needed to deep mine the influential genes in the genome.

**Results:**

Here, we propose a computational framework based on deep learning to mine a subset of genes that can cover more genomic information. Specifically, an AutoEncoder framework is first constructed to learn the non-linear relationship between genes, and then DeepLIFT is applied to calculate gene importance scores. Using this data-driven approach, we have re-obtained a landmark gene set. The result shows that our landmark genes can predict target genes more accurately and robustly than that of L1000 based on two metrics [mean absolute error (MAE) and Pearson correlation coefficient (PCC)]. This reveals that the landmark genes detected by our method contain more genomic information.

**Conclusions:**

We believe that our proposed framework is very suitable for the analysis of biological big data to reveal the mysteries of life. Furthermore, the landmark genes inferred from this study can be used for the explosive amplification of gene expression profiles to facilitate research into functional connections.

## Background

One of the fundamental challenges that has emerged throughout biomedicine is the need to establish relationships between disease, physiological processes and the role of small molecule therapies. To address this problem, a genomic signature is required that should have sufficiently high complexity to provide a rich description for all biological states, including those that are physiological, related to disease, or induced with a chemical, and that should be generated in a low-cost and high-throughput way. Gene expression profiling has been widely applied in medicine and biology to elucidate the response mechanism of cells to diseases, genetic interference and drug therapy [1, 2]; using this technique, the Connectivity Map (CMap) project has been proposed a systematic approach to discover functional connections among diseases, genetic perturbation, and drug action. Meanwhile, this study also suggested the value of a large-scale community CMap project [3].

Higher requirements have been put forward for the scale of the CMap project, and a diversity of chemical perturbations, genetic perturbations, and cell types await to be characterized. Unfortunately, although the price of commercial gene expression microarrays has been decreasing steadily, the high cost of profiling thousands of samples makes this prospect difficult. Therefore, how to reduce the cost of acquiring gene expression profiles is the first problem to be solved.

Previous studies have shown that although there are a large number of genes in the genome, most of their expression patterns are highly correlated [4, 5]. Cluster analysis of single-cell RNA-Seq indicated that genes from the same cluster showed similar expression patterns under different conditions [6]. Given such high similarity, researchers from the Library of Integrated Network-Based Cellular Signatures (LINCS) program hypothesized that it is possible to capture any cellular state at a low cost by measuring a reduced representation of the transcriptome [7]. Using Affymetrix HG-U133A microarray data from the Gene Expression Omnibus (GEO) [8], these researchers applied an iterative peel-off procedure based cluster analysis to identify the subset of universally informative transcripts termed ‘landmark genes’. According to the LINCS analysis, a set of ~ 1000 genes was finally identified as landmark genes, which was sufficient to recover 82% of the information in the full transcriptome. Then, the expression profile of the target genes was inferred by a linear regression algorithm, which was subsequently improved several times to improve the reliability of prediction [9, 10]. Finally, based on the ~ 1000 landmark genes, a new, low-cost, high-throughput reduced representation expression profiling method called L1000 was proposed, with which one million profiles were reported for the first time [7].

Cluster analysis mostly measures the similarity between variables by linear distance, such as Euclidean distance. As nonlinear regulatory relationships between genes are very common in biology [11], it is difficult for the ~ 1000 landmark genes inferred by cluster analysis to fully represent genomic information. Therefore, a new computational method with the capacity to capture the non-linear relationships of genes is needed to re-mine the influential genes that cover more information about the genome.

Deep learning, a non-linear network structure using multi-layer non-linear functions, has recently emerged based on big data, and academic interest has increased rapidly since the early 2000s [12]. Furthermore, the recent success of deep learning in diverse fields such as image and speech recognition [13, 14], natural language processing [15, 16], and bioinformatics [17, 18] suggests its ability to learn hierarchical nonlinear patterns on large data sets. Deep learning can be divided into supervised learning and unsupervised learning. The former mainly includes deep neural network (DNN), convolutional neural network (CNN) and recurrent neural network (RNN) and is mainly used for classification tasks such as transcription factor binding site prediction [19], promoter prediction [20] and predicting the effects of noncoding variants [21]. The most representative of the latter is AutoEncoder, which is commonly used for dimension reduction [22] to analyse high-dimensional gene expression data [23, 24] and to integrate heterogeneous data [25–27]. As a non-linear feature extraction method, AutoEncoder is capable of learning more useful features than linear feature extraction methods, such as principal component analysis (PCA).

Despite deep neural networks become increasingly popular, there is still a "black box" nature that hinders their application when interpretability is paramount. Understanding how an input feature affects a particular input can lead to new scientific discoveries. Therefore, multiple studies have been conducted to explain this “black box” [28–30]. Similarly, DeepLIFT is an efficient and effective method for computing importance scores in a neural network by comparing the activation of each neuron to a reference activation [31]. This method has been successfully applied to visualize splice site-related motifs from a trained CNN model [32].

Here, we present a deep learning framework to mine a gene set that can cover more genomic information. Specifically, we first constructed an AutoEncoder framework using ~ 130,000 gene expression profiles from the GEO Affymetrix microarray platform for training to learn the complex regulatory relationships across genes. Using this model, ~ 22,000 dimensional expression data were reduced to only 100. Clustering analysis of lung cancer showed that these 100 dimensional features well represent the biological information of gene expression data. Then, DeepLIFT was applied to measure the impact of each input layer neuron on the bottleneck layer neurons by providing an importance score. Using this data-driven approach, we obtained a list of genes that were sorted based on the importance score. By extracting genes from top to bottom, a new landmark gene set with the same number of genes as the original set from L1000 was finally identified. To compare the two landmark gene sets, we next used D-GEX [33] as a prediction model to infer the expression profiles of the target genes (besides the landmark genes) based on the landmark genes. The result shows that our landmark gene set can predict target genes more accurately and reliably than that of L1000 by comparing two performance metrics, MAE and PCC. Therefore, the landmark genes inferred by our method truly contain more information about the genome and are more suitable for expanding the scale of the CMap project.

## Results and discussion

### A brief summary of the computational framework

**Fig. 1:**
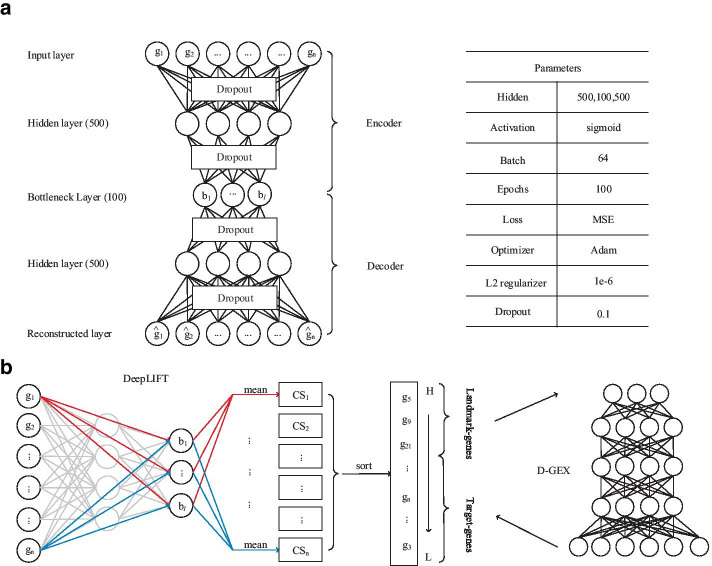
The workflow for mining influential genes based deep learning. **a** The architecture and parameter settings of AutoEncoder. **b** Application of DeepLIFT to compute the importance scores in the Encoder network and use of D-GEX as a baseline method to predict target genes for performance evaluation

Our computational framework mainly consists of two parts, AutoEncoder-based and DeepLIFT-based (Fig. 1, see “Methods” for details). In the AutoEncoder-based part, we use ~ 130,000 gene expression profiles to train an AutoEncoder that is composed of two steps, encoder and decoder. However, AutoEncoder is a feature extraction method that transforms data from the original, high-dimensional space to a relatively low-dimensional space. In other words, new features are generally different from original features. Here, the encoder compresses the 22,268 dimensional samples to 100 dimensions. In the DeepLIFT-based part, we use DeepLIFT to compute the importance scores of each input layer neuron on the bottleneck layer neurons. Then, we rank the genes based on the average importance scores, and the new landmark genes (see Additional file 1) can be identified by selecting the top 943 genes (the same number as the L1000).

### Performance evaluation of the AutoEncoder model

**Fig. 2:**
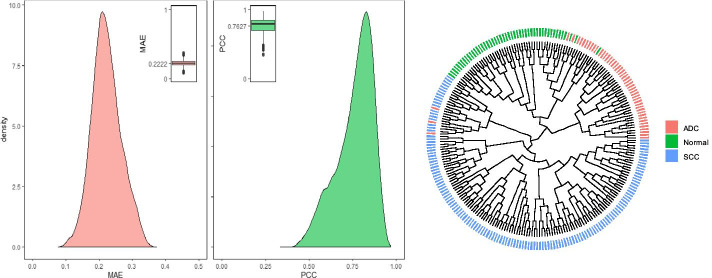
Performance evaluation of the AutoEncoder model in both gene (**a**) and sample dimensions (**b**). **a** The density plots of the predictive error (MAE) and the similarity (PCC) of all genes. **b** The circular diagram of clustering for three types of samples, including normal (Normal), lung adenocarcinoma (ADC) and lung squamous cell carcinoma (SCC)

After training the AutoEncoder model with GEO-based training samples (99,909), we use reserved test samples (11,100) to evaluate its predictive power in both gene and sample dimensions. In terms of genes, we use MAE and PCC to measure the prediction error and similarity of each gene. As shown in Fig. 2a, the average MAE and PCC of all genes are 0.2222 and 0.7627, respectively, and the permutation test shows that there is a significant high similarity between the predicted value and the real value of almost all genes (21,696/22,268). In terms of samples, we collect 237 lung cancer samples from the GEO database as new test samples, including 49 normal samples, 58 lung adenocarcinoma (ADC) samples and 130 lung squamous cell carcinoma (SCC) samples. Then, we take the expression profiles of these samples as the input of the trained AutoEncoder and use the output of the bottleneck layer to cluster the samples. Figure 2b shows that the low dimensional space mapped by the trained AutoEncoder well retains the biological information of the samples. All of these results show that our trained AutoEncoder can learn the non-linear relationships between genes well.

### Comparison of the landmark genes

**Fig. 3:**
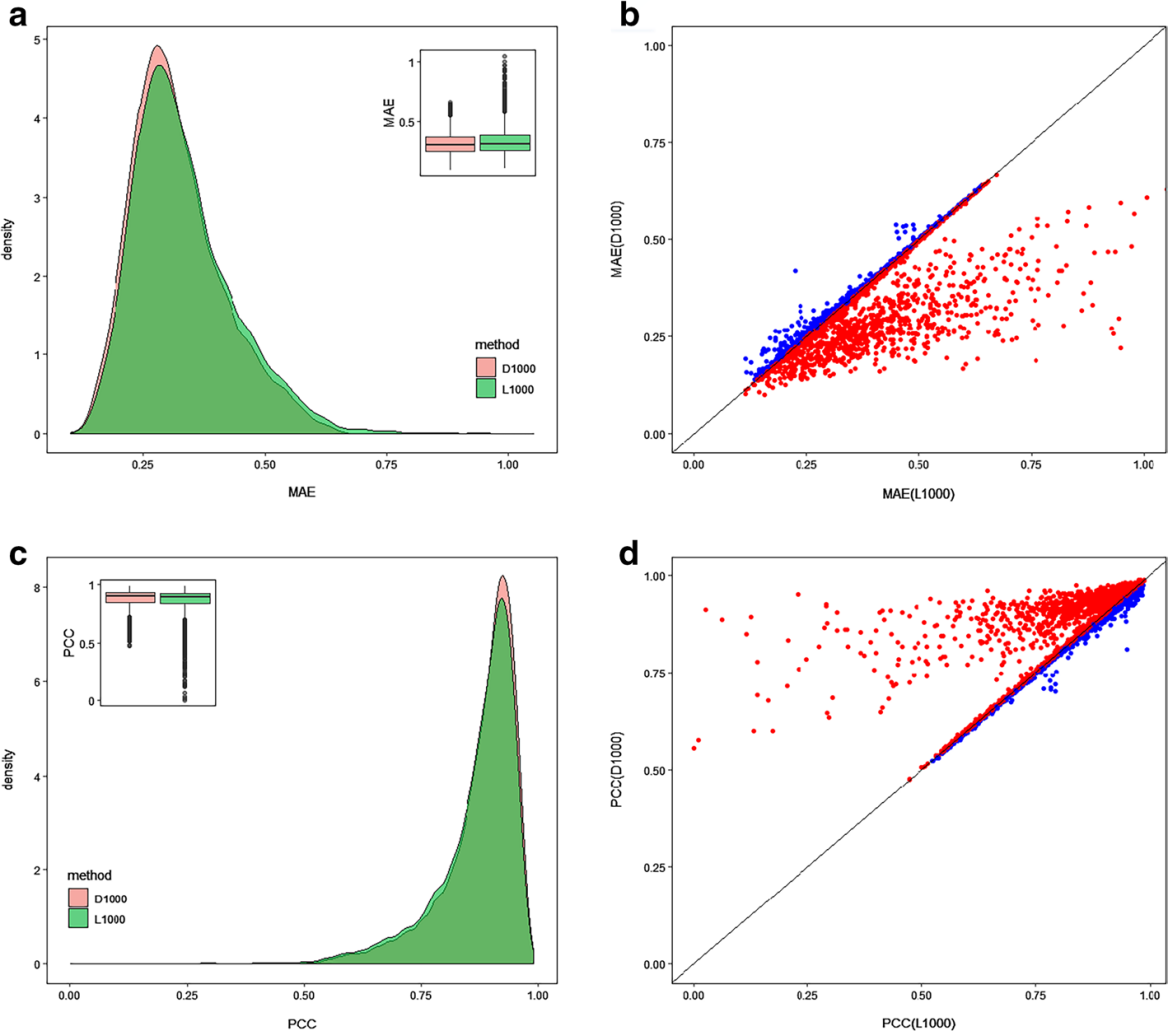
The density plot (**a**, **c**) and scatter plot (**b**, **d**) are used for comparison of the landmark genes inferred from our method (labelled as “D1000”) and that of L1000 (labelled as “L1000”) in terms of MAE (**a**, **b**) and PCC (**c**, **d**). In B and D, each dot represents a predicted target gene, and the red dot indicates that D1000 is better than L1000

First, we analyse the degree of overlap between our landmark genes (called D1000) and the landmark genes from L1000 (called L1000) and find that only 129 genes are shared. In addition, to evaluate the performance of the landmark genes inferred by our method, we use them as input to infer the expression profile of the target genes using a deep learn-based method, D-GEX. Then, we also use the MAE and PCC of each common target gene (9163) to compare D1000 with L1000. We define MAE and PCC of the target genes inferred from L1000 and D1000 as *MAE*_L1000_, *MAE*_D1000_, *PCC*_L1000_, and *PCC*_D1000_, respectively. As shown in Fig. 3a, b, compared with *MAE*_L1000_ with a value of 0.1129–1.0524, the *MAE*_D1000_value range is 0.0994–0.6681, and the paired t-test shows that *MAE*_L1000_ is significantly lower than*MAE*_L1000_ (p < 0.01). Similarly, as shown in Fig. 3c, d, compared with *PCC*_L1000_ with a value of 0.0006–0.9875, the*PCC*_D1000_value range is 0.4764–0.9905, and the paired t-test shows that *PCC*_D1000_is significantly higher than*PCC*_L1000_ (p < 0.01). Furthermore, all*PCC*_D1000_pass the permutation test, but 44 target genes fail in*PCC*_L1000_. These results show that the new landmark genes inferred from our method can predict target genes more accurately and robustly than the old landmark genes.

### Cross-platform generalization analysis of the landmark genes

**Fig. 4:**
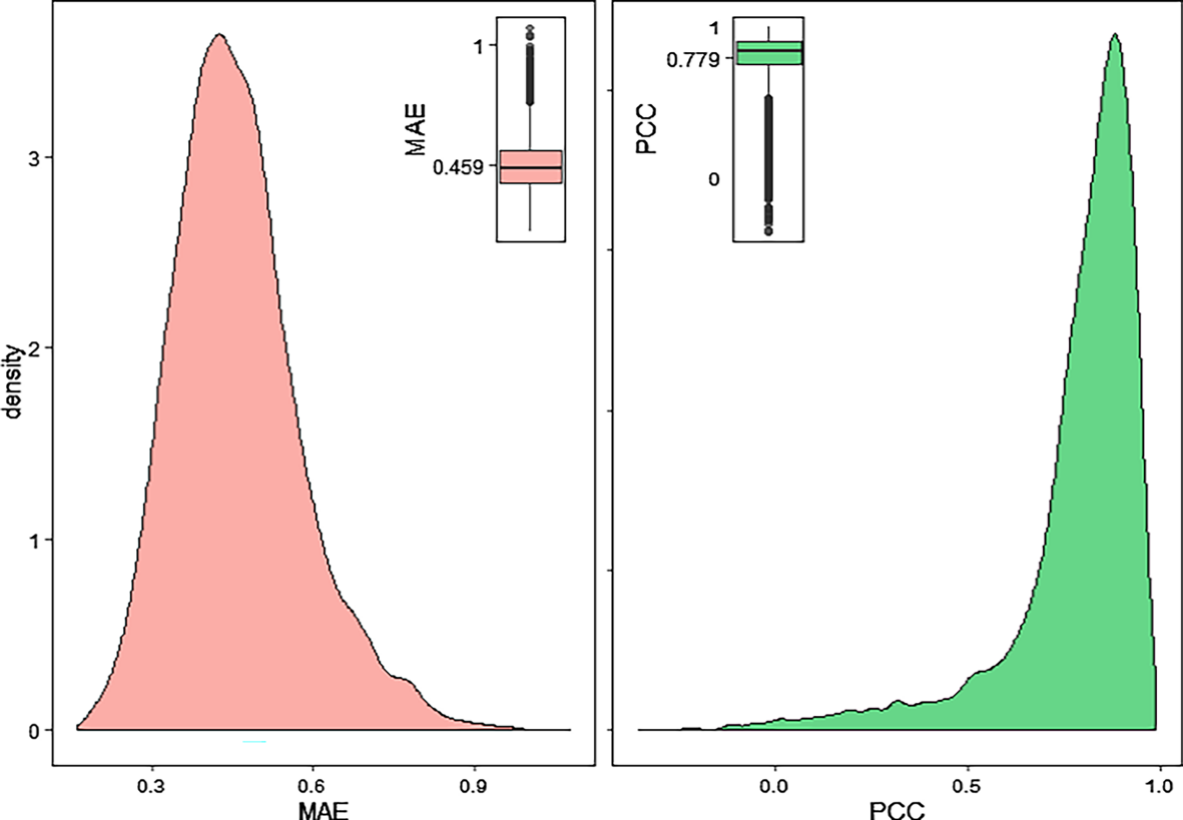
Cross-platform generalization analysis of the landmark genes inferred from our method

RNA-Seq is another high-throughput sequencing platform that has gradually become the standard for gene expression profiling. Next, to explore the ability to use landmark genes inferred from the microarray-based GEO dataset to infer target genes from the RNA-Seq-based expression profiling, we download a RNA-Seq-based gene expression profiling containing 2921 samples from GTEx database, and the predicted target genes are analysed. The results indicate that the average MAE and PCC of all target genes are 0.4590 and 0.7790 (Fig. 4), respectively, and that 92.51% of the target genes pass the permutation test, which shows that the landmark genes have excellent cross-platform generalization.

### Functional analysis of the landmark genes

Finally, to analyse whether the landmark genes suggested by our data-driven approach based on the analysis of 129,158 samples are enriched in particular known biological categories, we study their molecular functions from the perspective of Gene Ontology (GO). Given that the landmark genes cover most information about the genome, we infer that the landmark genes, when considered as a set, are dominated by either very few functions or many functions.

**Fig. 5:**
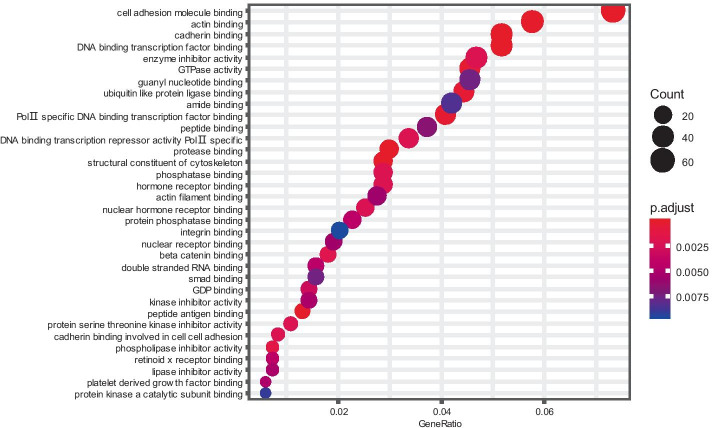
Enriched GO molecular functions term by using the landmark genes as a set

To test this inference, we use the R Bioconductor package clusterProfiler (v3.10.1) to apply hypergeometric statistics between the 943 landmark genes and a database of 1,645 gene sets that come from molecular function terms compiled in Gene Ontology. As shown in Fig. 5, we observe only 34 functional categories, most of which tend to be basic and generic, such as “DNA binding transcription factor binding”, "GDP binding", "enzyme inhibitor activity" and "protease binding", and contain only a small fraction of the landmark genes (e.g., "cell adhesion molecule binding" contains 61 of 943 landmarks). The results show that no particular functional category dominates the landmark genes.

## Conclusion

The central dogma of molecular biology states that the flow of genetic information is "DNA to RNA to protein". Current biological studies, such as genomic studies including variable splicing and single nucleotide polymorphisms, and epigenomic studies including methylation and histone modification, are all ultimately concerned with the regulation of gene expression. Therefore, gene expression patterns can reflect almost every aspect of life activities and can be used as genomic signatures to discover the functional connections among diseases, genetic perturbation, and drug action.

In this study, we proposed a deep learning-based method to detect influential genes in the genome to obtain large-scale expression profiles at lower costs. In a nutshell, this is a question of feature selection. The computing framework we designed combines AutoEncoder and DeepLIFT to assess the impact of each gene in the genome. The novelty of our method comes from (1) the use of a data-driven approach in an unbiased manner rather than selecting transcripts based on prior biological knowledge; (2) features are filtered out through a computational framework that includes a nonlinear feature extraction method AutoEncoder and a feature scoring algorithm DeepLIFT. The results show that using our landmark gene set can predict target genes more accurately and robustly than the gene set inferred from cluster analysis and reflects the advantages of deep learning in nonlinear computation.

In general, we believe that the method proposed in this paper has two main contributions. Firstly, the calculation framework of Autoencoder combined with DeepLIFT can sort the dimensions by capturing the nonlinear relationship between the dimensions of input samples, which provides an idea for solving the problem of feature selection. Then, the benchmark genes obtained by our method can be used to establish large-scale compendia of the cellular effects of genetic perturbation in a low-cost and more accurate way, which lays the foundation for the subsequent discovery of the mechanism of action of small molecules, functionally annotate genetic variants of disease genes, and inform clinical trials.

## Methods

In this study, our goal is to extract ~ 1000 influential genes from ~ 22,000 genes, which is a feature selection problem. Although many feature selection methods such as subset selection [34] and random forest35, which are usually used in classification tasks, can effectively filter out redundant features, they cannot effectively capture the nonlinear relationship between features. In view of the above problems, we designed a computational framework as follows.

### Data sources

In Table 1, three publicly available datasets are used for our analysis: the microarray-based GEO dataset, the RNA-Seq-based GTEx dataset and the lung cancer subtype dataset. The first two were downloaded from https://cbcl.ics.uci.edu/public_data/D-GEX/; the latter, from the GEO database.

**Table 1:** Three expression datasets from the GEO and GTEx databases

Dataset	Sample size	Platform	Database
1	111,009	Microarray	GEO
2	2,921	RNA-Seq	GTEx
3	237	Microarray	GEO

First, the microarray-based GEO dataset is used to train AutoEncoder. This dataset contains 129,158 gene expression profiles, each of which contains 22,268 probes corresponding to 978 landmark genes and 21,290 target genes. The original expression data are quantile normalized to a range of values between 4 and 15 to remove technical variation[36]. Considering that a dataset containing a large number of redundant samples with high similarity corresponds to low statistical representativeness[37], the k-means clustering program is used to remove duplicated profiles. Finally, the remaining 111,009 samples are randomly divided into ~ 90% (99,909) for training and ~ 10% (11,100) for testing.

Next, cross-platform performance can be evaluated based on the RNA-Seq dataset from GTEx, which contains 2,921 gene expression profiles of various tissue samples produced on RNA-Seq platform in the format of reads per kilobase per million (RPKM). We refer to the pre-processing protocol used in D-GEX for cross-platform data matching and joint quantile normalization. The 22,268 probes are finally matched to 10,463 genes based on Gencode V12 annotations, including 943 landmark genes and 9520 target genes.

Finally, the lung cancer subtype dataset is used to verify whether AutoEncoder can effectively learn biological information. This dataset contains 237 gene expression profiles from the GSE4573 and GSE10072 microarray datasets, including 49 normal samples, 58 lung adenocarcinoma (ADC) samples and 130 lung squamous cell carcinoma (SCC) samples.

### AutoEncoder

AutoEncoder is a multi-task unsupervised feed-forward neural network with multiple stacked hidden layers, which is composed of two parts, an encoder and a decoder (Fig. 1a). Considering a dataset*X*with m samples and n features, the encoder${\left. E \right|_{X \to Y}}$aims to map the original data *X* to the reduced representation *Y* through the bottleneck layer, and the purpose of the decoder ${\left. D \right|_{Y \to X}}$is tuned to reconstruct the original data *X* from the low-dimensional representation *Y* by minimizing the difference between*X* and $\hat X$.

Specifically, we use the Python Keras library to implement an AutoEncoder with three hidden layers of 500, 100, and 500 nodes. For a given layer *l*, we use sigmoid as the activation function.

$$o = {f_1}\left( x \right) = sigmoid\left( {{w_1}x + {b_l}} \right)$$

where *x* is an input vector of size *d*, *w*_*l*_ is the weight matrix of size *p* × *d*, and *b*_*l*_ is an intercept vector of size *p*. Given a set of gene expression profilescontaining *m* samples, wheredenotes each gene expression profile containing *n* genes, the input vector is reconstructed to${\hat S_m}$ through a series of matrix transformations of multiple network layers. Training an AutoEncoder involves finding parameters $\theta = \left( {w,b} \right)$ minimizing a specific loss function. Here, we use Mean Absolute Error (MAE) as the loss function.

$$MAE(g,\hat g) = \frac{1}{n}\sum\limits_{i = 1}^n {\left| {{g_i} - {{\hat g}_i}} \right|}$$

To control overfitting, we add an L2 regularization penalty α =1e−6 on the weight vector. Thus, the loss function above becomes:

$$loss(g,\hat g) = \frac{1}{n}\sum\limits_{i = 1}^n {\left| {{g_i} - {{\hat g}_i}} \right|} + \sum\limits_{j = 1}^k {\alpha \left\| {W_j} \right\|}_2^2$$

Finally, AutoEncoder is trained using the Adam [38] optimization algorithm with 100 epochs and 10% dropout.

### DeepLIFT

DeepLIFT is a feature scoring algorithm, which calculating contribution scores by comparing the activation of each neuron to its ‘reference activation’ [30]. In contrast to most gradient-based methods, using a difference-from-reference allows DeepLIFT to propagate an importance signal even in situations where the gradient is zero and avoids artifacts caused by discontinuities in the gradient.

In our computing framework, for every gene of input samples, a contribution score is firstly calculated by making use of the Rescale Rule of the DeepLIFT algorithm. The obtained contribution scores express the importance of the corresponding genes for the compression features of the bottleneck layer. Then, we rank the genes based on the importance scores, and the new landmark genes (see Additional file 1) can be identified by selecting the top 943 genes (the same number as the L1000). For more details on the usage of DeepLIFT, we would like to refer the interested reader to reference [30].

### D-GEX

D-GEX model is a deep learning method to infer the expression of target genes from the expression of landmark genes [9]. To test the reliability of the landmark genes derived from the AutoEncoder combined with DeepLIFT method, we use D-GEX model to compare the ability of the landmark genes to infer target gene expression with the L1000 method. In our study, we used the default parameters of D-GEX.

### Evaluation metrics

Given a test set = (*s*_1…,*Sm*_)containing samples, we use two different metrics for the evaluation of predicted expression. For each gene *g*_*j*_, the definition of MAE is:

$$MA{E_j} = \frac{1}{m}\sum\limits_{i = 1}^m {\left| {{g_{ij}} - {{\hat g}_{ij}}} \right|}$$

The following equation shows the definition of PCC:

$$PC{C_j} = \rho \left( {{g_j},{{\hat g}_j}} \right) = \frac{{\sum\limits_{i = 1}^m {\left( {{g_{ij}} - {\mu_j}} \right)\left( {{{\hat g}_{ij}} - {{\hat \mu }_j}} \right)} }}{{\sqrt {{{\left( {{g_{ij}} - {\mu_j}} \right)}^2}} {{\sqrt {\left( {{{\hat g}_{ij}} - {{\hat \mu }_j}} \right)} }^2}}}$$

where indicates the Pearson correlation coefficient for the *j*-th predicted gene and ${\mu_j},{\hat \mu_j}$ are the mean of ${g_j},{\hat g_j}$respectively.

The Pearson correlation coefficient, an absolute measure of similarity between genes, does not in itself reflect how uncommon that similarity is. Hence, we apply a permutation test to aid in the interpretation of similarity. Briefly, in addition to computing thebetween ${g_j}\;{\text{and}}\;{\hat g_j}$, we also compute thebetween the${\hat g_j}$and any gene other thanas a reference distribution of similarity values. After that, we compareto, and if the fraction of that is higher than is lower than 0.01, ${g_j}\;{\text{and}}\;{\hat g_j}$are considered to be significantly correlated.

## Supplementary Information

**Additional file 1.** A file in TXT format including a list of genes sorted based on contribution scores.

## Data Availability

Three publicly available datasets are used for our analysis: the microarray-based GEO dataset, the RNA-Seq-based GTEx dataset and the lung cancer subtype dataset. The first two were downloaded from https://cbcl.ics.uci.edu/public_data/D-GEX/; the latter, from the GEO database with GSE4573 and GSE10072.

## Abbreviations

CMap: Connectivity map
MAE: Mean absolute error
PCC: Pearson correlation coefficient
GEO: Gene expression omnibus
ADC: Lung adenocarcinoma
SCC: Lung squamous cell carcinoma
